# Supplementary material for: Grain structure control during metal 3D printing by high-intensity ultrasound
Source: Nat Commun. 2020 Jan 9;11:142. doi: 10.1038/s41467-019-13874-z (PMC6952421; doi:10.1038/s41467-019-13874-z)
Supplement: Supplementary file 1 — Supplementary information [file 41467_2019_13874_MOESM1_ESM.pdf]

Grain structure control during metal 3D printing by high-intensity ultrasound

Todaro et al.

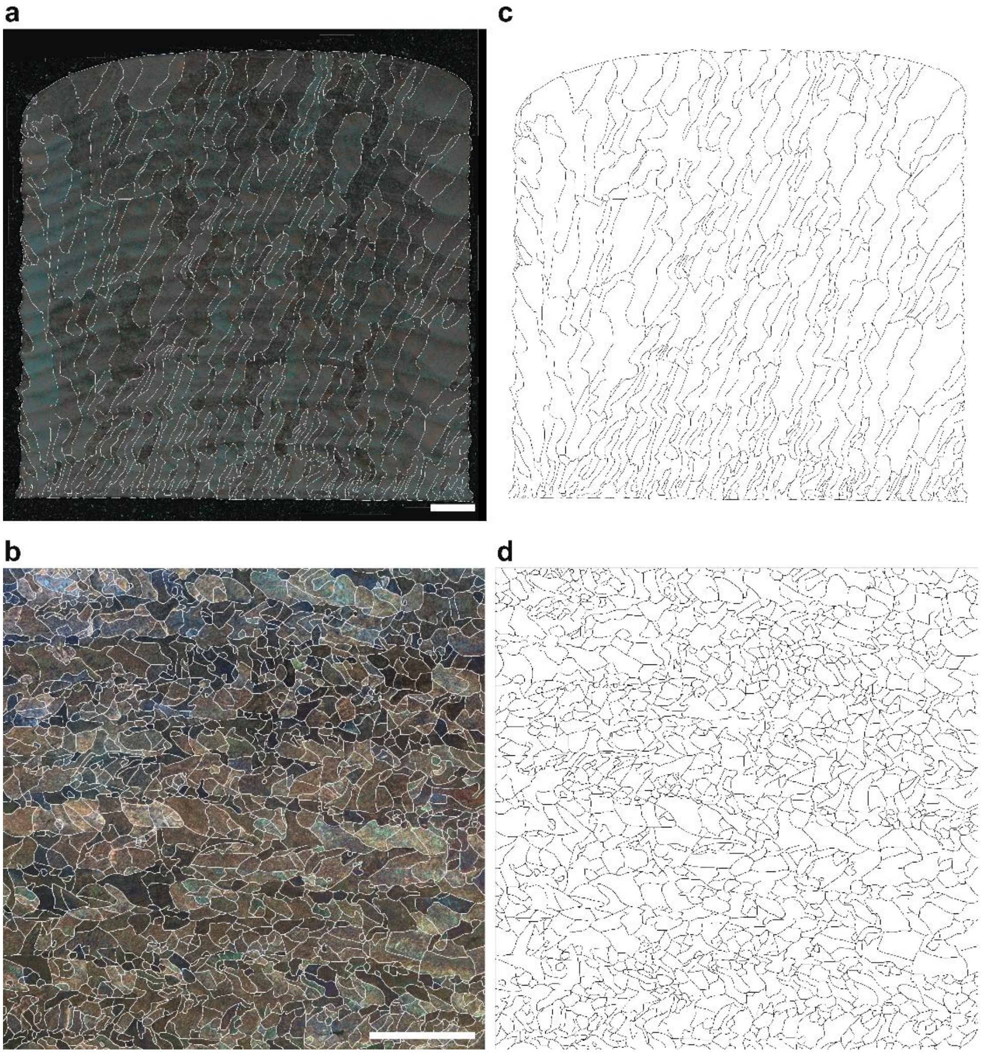

Supplementary Fig. 1 Images of traced prior-β grains in the AM-fabricated Ti-6Al-4V samples. **a**, **b** Polarized light microscopy images of samples **a** without and **c** with ultrasound. The prior-β grain boundaries are traced in white. **c**, **d** The corresponding images of traced prior-β grains used to determine the prior-β grain size and prior-β grain aspect ratio distributions. Scale bars, 1 mm.

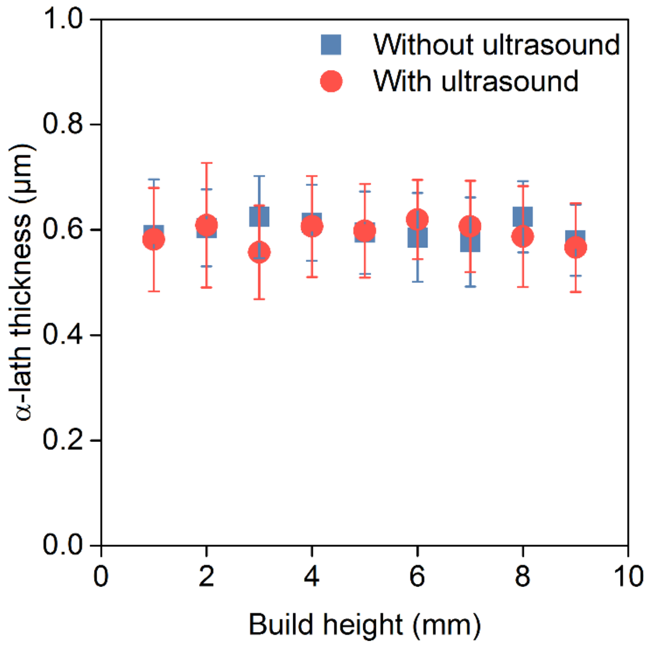

Supplementary Fig. 2 α-lath thickness of the AM-fabricated Ti-6Al-4V samples without and with high-intensity ultrasound versus build height. No statistical difference in α-lath thickness is found between the two samples and at the different build heights in each sample. The error bars represent one standard deviation of ~40 line intercept measurements from three SEM images per data point.

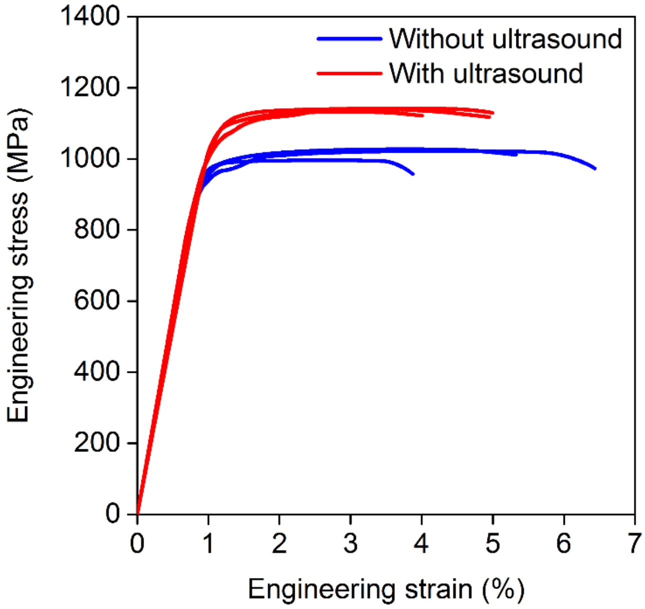

Supplementary Fig. 3 Tensile engineering stress-strain curves of the AM-fabricated as-built Ti-6Al-4V samples without and with high-intensity ultrasound.

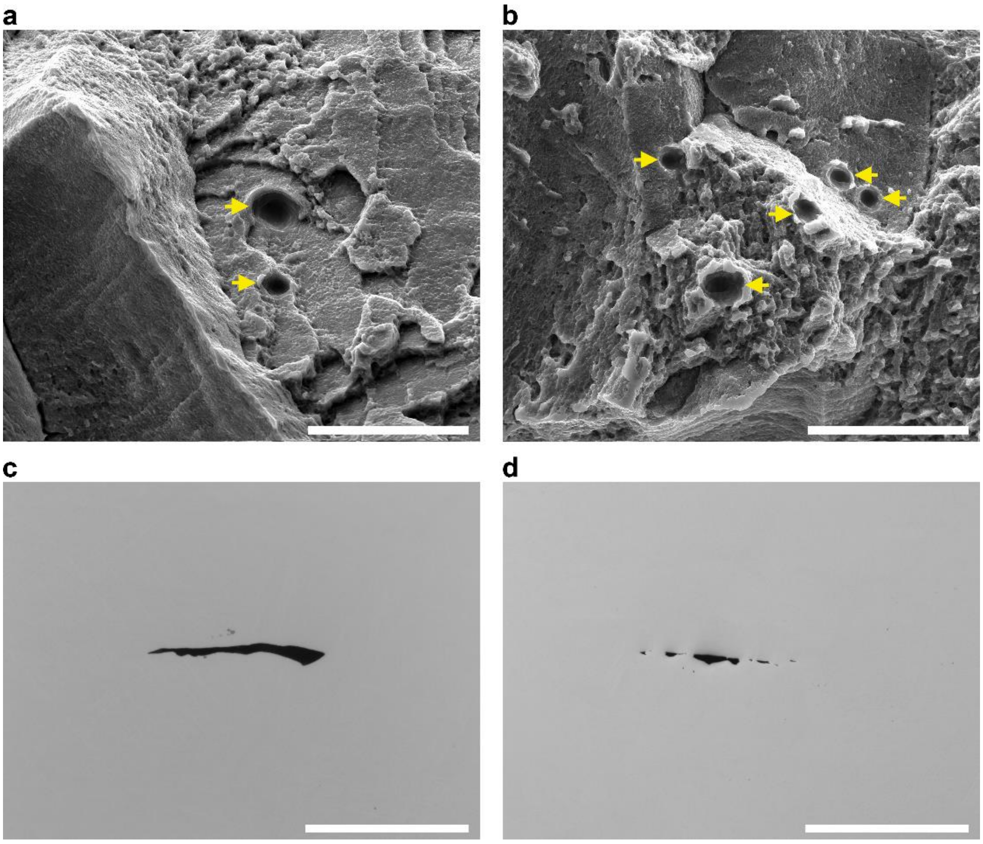

Supplementary Fig. 4 Typical defects in the AM-fabricated Ti-6Al-4V samples. **a**, **b** SEM images showing isolated pores (arrowed) on the fracture surfaces of samples **a** without and **b** with high-intensity ultrasound. **c**, **d** Optical microscopy images showing ~200 µm wide lack-of-fusion defects on the polished cross-sections of the samples **c** without and **d** with ultrasound. Scale bars, 50 µm in **a**, **b**, 200 µm in **c**, **d**.

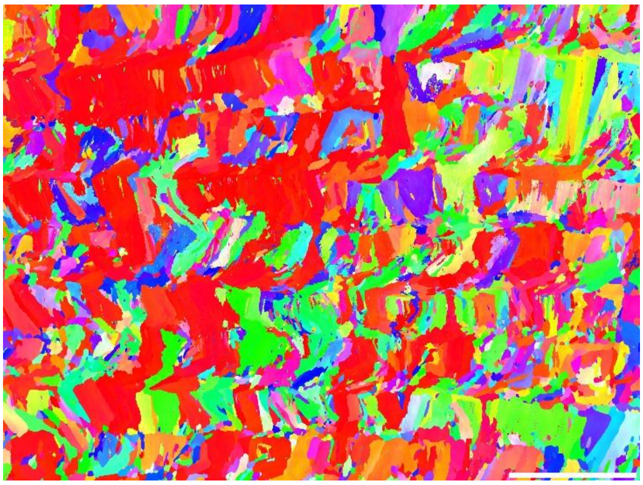

Supplementary Fig. 5 A low magnification inverse pole figure map along the build direction (*z*) of the AM-fabricated Inconel 625 sample without high-intensity ultrasound. This data set was used to plot the {001} contoured pole figure in Fig. 6c. Scale bar, 1000 μm.

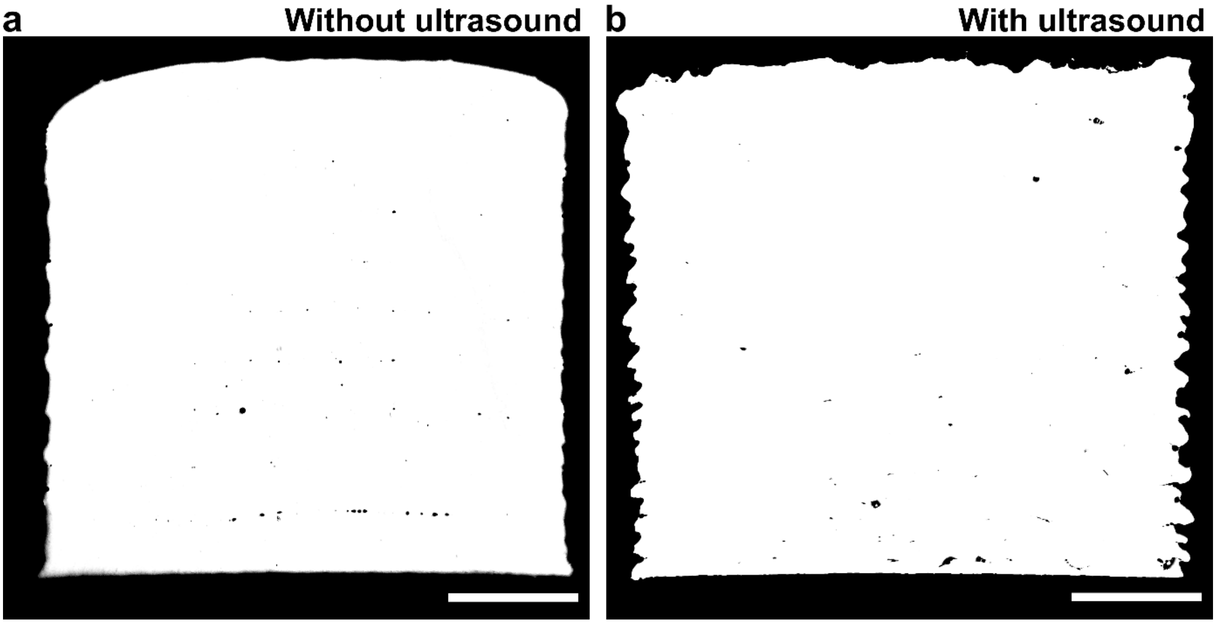

Supplementary Fig. 6 The effect of ultrasound on the porosity in the AM-fabricated Ti-6Al-4V samples. **a**, **b** Optical microscopy images of the polished cross-sections of the samples **a** without and **b** with ultrasound. The porosity in both samples is ~0.7-0.9 area%. Scale bars, 250 µm.

Supplementary Table 1 The average tensile properties of the AM-fabricated as-built Ti-6Al-4V samples without and with high-intensity ultrasound. The error values represent one standard deviation of three tests.

**Sample**	**Yield stress, 0.2% (MPa)**	**Tensile strength (MPa)**	**Strain to failure (%)**
Without ultrasound	980 ± 13	1015 ± 14	5.1 ± 0.9
With ultrasound	1094 ± 18	1137 ± 4	4.7 ± 0.4

Supplementary Table 2 Compositions of the AM-fabricated Ti-6Al-4V samples without and with high-intensity ultrasound determined by ICP-AES and LECO combustion.

**Sample**	**Composition (wt%)**								
	Ti	Al	V	Fe	Cr	O	N	C	H
Without ultrasound	Bal.	6.01	4.02	0.19	0.01	0.15	0.011	0.01	<0.005
With ultrasound	Bal.	6.02	3.98	0.27	0.02	0.14	0.012	0.01	<0.005

Supplementary Table 3 Change in yield stress of AM-fabricated Ti-6Al-4V by chemical addition compared to high-intensity ultrasound in this work.

Sample	Process	Yield stress, 0.2% (MPa)	Change in yield stress, 0.2% (%)	Ref.
Ti-6Al-4V	Arc-based wire deposition	800	-	[1]
Ti-6Al-4V-0.05B		805	0.6	
Ti-6Al-4V	Arc-based wire deposition	800	-	[1]
Ti-6Al-4V-0.11LaB_6		850	6.3	
Ti-6Al-4V	Arc-based wire deposition	760	-	[2]
Ti-6Al-4V-0.03C		805	5.9	
Ti-6Al-4V-0.1C		830	9.2	
Ti-6Al-4V-0.41C		830	9.2	
Ti-6Al-4V (without ultrasound)	Laser-based directed energy deposition	980	-	This work
Ti-6Al-4V (with ultrasound)		1094	11.6	

Supplementary Table 4 Comparison of the prior-β grain size, α-lath thickness and yield stress of AM-fabricated α-β (basketweave-like) Ti-6Al-4V. The orientation of the tensile samples is normal to the build direction. The prior-β grain size is given as the prior-β grain width. NA: not available.

Sample condition	AM process	Prior-β grain size (μm)	α-lath thickness (μm)	Yield stress, 0.2% (MPa)	Ref.
As-built	Electron beam-based powder bed fusion	~100	1.9	1024	[3]
As-built	Laser-based powder bed fusion	103	0.570	978	[4]
As-built		103	0.570	1075	
Stress relief		103	1.2	958	
Stress relief		103	1.2	974	
As-built	Laser-based directed energy deposition	375	~0.915	960	[5]
Solution treatment and aging	Laser-based directed energy deposition	1000	3.3	791.6	[6]
		630	1.5	839.5	
Low temperature homogenization	Laser-based powder bed fusion	150	1	965	[7]

High temperature homogenization		150	2.3	944	
Stress relief	Arc-based wire deposition	~1400	~1.5	766	[8]
Solution treatment and aging		~1400	~0.72	858	
Vacuum heat treatment		~1400	~5.9	721	
Hot isostatic pressing		~1400	~7.1	712	
As-built	Laser-based powder bed fusion	~150	NA	910	[9]
As-built	Laser-based directed energy deposition	~310	NA	950	[10]
As-built	Laser-based powder bed fusion	~125	0.3	1106	[11]
As-built	Laser-based powder bed fusion	70	0.52	1022	[12]
Stress relief	Laser-based powder bed fusion	96.3	NA	1141	[13]
		190.2	NA	1010	
As-built	Laser-based directed energy deposition	300	0.60	980	This work
As-built		117	0.59	1094	

Supplementary Note 1

Cavitation during additive manufacturing of Ti-6Al-4V

The cavitation threshold is determined by the surface tension of the melt. For molten Ti-6Al-4V, its surface tension (σ) above the liquidus temperature (1655 °C) has been systematically measured and obeys the following relationship with melt temperature (T) [14]:

$$\sigma = 1.52 - (T - 1655) \times (5.52 \times 10^{-4}) \text{ (in N m}^{-1}\text{)} \quad (1)$$

The melt pool temperature of Ti-6Al-4V during directed energy deposition has been measured and the temperature at the centre can reach 2500 °C [15]. Accordingly, the surface tension of the Ti-6Al-4V melt during AM is about 1.05 N m^{-1}.

Measuring cavitation in molten metals is challenging and we are not aware of any experimental measurement in molten Ti and Ti alloys. However, Eskin has systematically measured cavitation in commercially pure molten Al [16]. The surface tension of molten Al at 710 °C was measured to be ~0.9 N m^{-1} [17], which is similar to the surface tension of molten Ti-6Al-4V (1.05 N m^{-1}) in the melt pool during directed energy deposition. The ultrasonic conditions used in this study (ultrasonic amplitude: 30 µm, frequency: 20 kHz) are included in those used by Eskin [16]. Eskin [16] investigated the effect of ultrasonic amplitude (2, 5, 10, 15, 20, 30 and 40 µm) on cavitation in molten Al and identified that cavitation was incipient when the amplitude was increased from 2 µm to 5 µm and became fully developed when the amplitude reached 10 µm and beyond. As shown by Equation (1) in the manuscript, the ultrasonic intensity is proportional to the square of amplitude (A). The 30-µm amplitude applied in this study is 9 times the ultrasonic intensity required for the generation of fully developed cavitation in molten Al. As pointed out earlier, the surface tension of molten Ti-6Al-4V in this study is similar to that of the molten Al investigated by Eskin [16]. In other words, the ultrasonic conditions employed in this study are well above the threshold required for cavitation in molten Ti-6Al-4V during directed energy deposition.

Kinetically, the directed energy deposition process used in this study offers far more than sufficient time for cavitation to occur in the melt pool. The laser beam size used was 0.61 mm. The melt pool size is typically about 30% larger than the beam size, which gives about 0.8 mm. The laser scanning speed used was 600 mm min^{-1}. The melt pool survival time is equivalent to the time for the laser to travel across the melt pool, which is 0.08 s. On the other hand, the time required from bubble formation to bubble implosion in liquid metals is ~30 μs (0.00003 s), measured by in situ ultrafast high-resolution synchrotron X-ray imaging [18]. Hence, the melt pool survival time is far more than sufficient for cavitation to occur in the melt pool.

Supplementary references

1. M. J. Bermingham, S. D. McDonald and M. S. Dargusch: *Mater. Sci. Eng. A,* 2018, vol. 719, pp. 1-11.
2. S. Mereddy, M. J. Bermingham, D. Kent, A. Dehghan-Manshadi, D. H. StJohn and M. S. Dargusch: *JOM,* 2018, vol. 70, pp. 1670-76.
3. C. de Formanoir, S. Michotte, O. Rigo, L. Germain and S. Godet: *Mater. Sci. Eng. A,* 2016, vol. 652, pp. 105-19.
4. M. Simonelli, Y. Y. Tse and C. Tuck: *Mater. Sci. Eng. A,* 2014, vol. 616, pp. 1-11.
5. B. E. Carroll, T. A. Palmer and A. M. Beese: *Acta Mater.,* 2015, vol. 87, pp. 309-20.
6. Y. M. Ren, X. Lin, X. Fu, H. Tan, J. Chen and W. D. Huang: *Acta Mater.,* 2017, vol. 132, pp. 82-95.
7. T. Vilaro, C. Colin and J. D. Bartout: *Metall. Mater. Trans. A,* 2011, vol. 42a, pp. 3190-99.
8. M. J. Bermingham, L. Nicastro, D. Kent, Y. Chen and M. S. Dargusch: *J. Alloy. Comp.,* 2018, vol. 753, pp. 247-55.
9. P. Edwards and M. Ramulu: *Mater. Sci. Eng. A,* 2014, vol. 598, pp. 327-37.
10. C. Qiu, G. A. Ravi, C. Dance, A. Ranson, S. Dilworth and M. M. Attallah: *J. Alloy. Comp.,* 2015, vol. 629, pp. 351-61.
11. W. Xu, M. Brandt, S. Sun, J. Elambasseril, Q. Liu, K. Latham, K. Xia and M. Qian: *Acta Mater.,* 2015, vol. 85, pp. 74-84.
12. W. Xu, E. W. Lui, A. Pateras, M. Qian and M. Brandt: *Acta Mater.,* 2017, vol. 125, pp. 390-400.
13. A. E. Wilson-Heid, Z. Wang, B. McCornac and A. M. Beese: *Mater. Sci. Eng. A,* 2017, vol. 706, pp. 287-94.
14. R. Aune, L. Battezzati, R. Brooks, I. Egry, H. J. Fecht, J. P. Garandet, K. C. Mills, A Passerone, P. N. Quested, E. Ricci, S. Schneider, S. Seetharaman, R. K. Wunderlich and B. Vinet: *Microgravity Sci. Technol.,* 2005, vol. 16, pp. 11-14.
15. G. J. Marshall, W. J. Young, S. M. Thompson, N. Shamsaei, S. R. Daniewicz and S. Shao: *JOM,* 2016, vol. 68, pp. 778-90.
16. G. I. Eskin and D. G. Eskin: *Ultrasonic Treatment of Light Alloy Melts,* 2nd ed., CRC Press, Boca Raton, 2014.
17. I. F. Bainbridge and J. A. Taylor: *Metall. Mater. Trans. A,* 2013, vol. 44a, pp. 3901-09.
18. B. Wang, D. Y. Tan, T. L. Lee, J. C. Khong, F. Wang, D. Eskin, T. Connolley, K. Fezzaa and J. W. Mi: *Acta Mater.,* 2018, vol. 144, pp. 505-15.
